# Risk score based on three mRNA expression predicts the survival of bladder cancer

**DOI:** 10.18632/oncotarget.18642

**Published:** 2017-06-27

**Authors:** Qingzuo Liu, Ruigang Diao, Guoyan Feng, Xiaodong Mu, Aiqun Li

**Affiliations:** ^1^ Yantai Yuhuangding Hospital, Zhifu District, Yantai 264000, China; ^2^ Yantai Affiliated Hospital of Binzhou Medical University, Muping District, Yantai 264003, China

**Keywords:** bladder cancer, prognosis, expression

## Abstract

Bladder cancer (BLCA) is one of the most malignant cancers worldwide, and its prognosis varies. 1214 BLCA samples in five different datasets and 2 platforms were enrolled in this study. By utilizing the gene expression in The Cancer Genome Atlas (TCGA) dataset, and another two datasets, in GSE13507 and GSE31684, we constructed a risk score staging system with Cox multivariate regression to evaluate predict the outcome of BLCA patients. Three genes consist of RCOR1, ST3GAL5, and COL10A1 were used to predict the survival of BLCA patients. The patients with low risk score have a better survival rate than those with high risk score, significantly. The survival profiles of another two datasets (GSE13507 and GSE31684), which were used for candidate gene selection, were similar as the training dataset (TCGA). Furthermore, survival prediction effect of risk score staging system in another 2 independent datasets, GSE40875 and E-TABM-4321, were also validated. Compared with other clinical observations, and the risk score performs better in evaluating the survival of BLCA patients. Moreover, the correlation between radiation were also evaluated, and we found that patients have a poor survival in high risk group, regardless of radiation. Gene Set Enrichment Analysis was also implemented to find the difference between high-risk and low-risk groups on biological pathways, and focal adhesion and JAK signaling pathway were significantly enriched. In summary, we developed a risk staging model for BLCA patients with three gene expression. The model is independent from and performs better than other clinical information.

## INTRODUCTION

Bladder cancer (BLCA) is one of the most malignant diseases worldwide, with 73,510 new cases and 14,880 deaths [1] in the United States, 2012. According to most recent statistic report in China, there were 80,500 new BLCA cases and 32,900 deaths occurred due to BLCA [2]. Although the improvement of the therapy methods and drugs, a large proportion of patients died within 3 years after diagnosis, which, makes the prognosis of BLCA important [3]. However, as most frequently used prognostic indicators, clinical observations often fail to predict the survival of bladder cancer patients. Thus, the molecular biomarkers are now urgently needed.

Based on previous studies, the performance of single biomarkers in predicting the survival of BLCA patients across datasets are unstable, while combination of biomarkers enhances the performance [4]. In this vein, we implemented Cox multivariate regression model on gene expression of BLCA samples in TCGA dataset. The patients with high risk score had a significantly shorter survival time than those with low risk score, and this finding was further validated in other two cohorts used for candidate gene selection (GSE13507 and GSE31684) and another two totally independent datasets (GSE40875 and E-TABM-4321). Furthermore, according to cox multivariate hazard analyses, the risk score performs better than the other clinical information in prognosis of BLCA patients. The risk score is also effective in estimating the survival of patients whether they underwent radiation or not. Gene Set Enrichment Analysis (GSEA) showed that focal adhesion pathway was significantly altered between high and low risk group, suggesting that the risk score reflects the cell adhesion status of BLCA.

## RESULTS

### Identification of survival-related genes

With univariate Cox regression model, genes were used to evaluate the correlation between gene expression and overall survival in three independent datasets (TCGA-BLCA, GSE13507 and GSE31684). In order to improve the robustness of the candidate gens, mRNA levels significantly correlated with overall survival in all these three datasets (p<0.05) were selected for further analysis, and three genes, RCOR1, ST3GAL5 and COL10A1, were identified. Multivariate cox regression analyses were performed and the risk scores were calculated as the following:

Risk score=0.14738*RCOR1+(−0.17272)*ST3GAL5+0.18195*COL10A1.

It is noticed that the coefficient of ST3GAL5 is negative, indicating that the expression of this gene is positively related the survival time/rate of BLCA patients while the expression of RCOR1 and COL10A1 are negatively related. Detailed correlation information between overall survival information and the three gene expression was listed in Supplementary Table 1.

### Performance of risk score in training dataset

**Figure 1:**
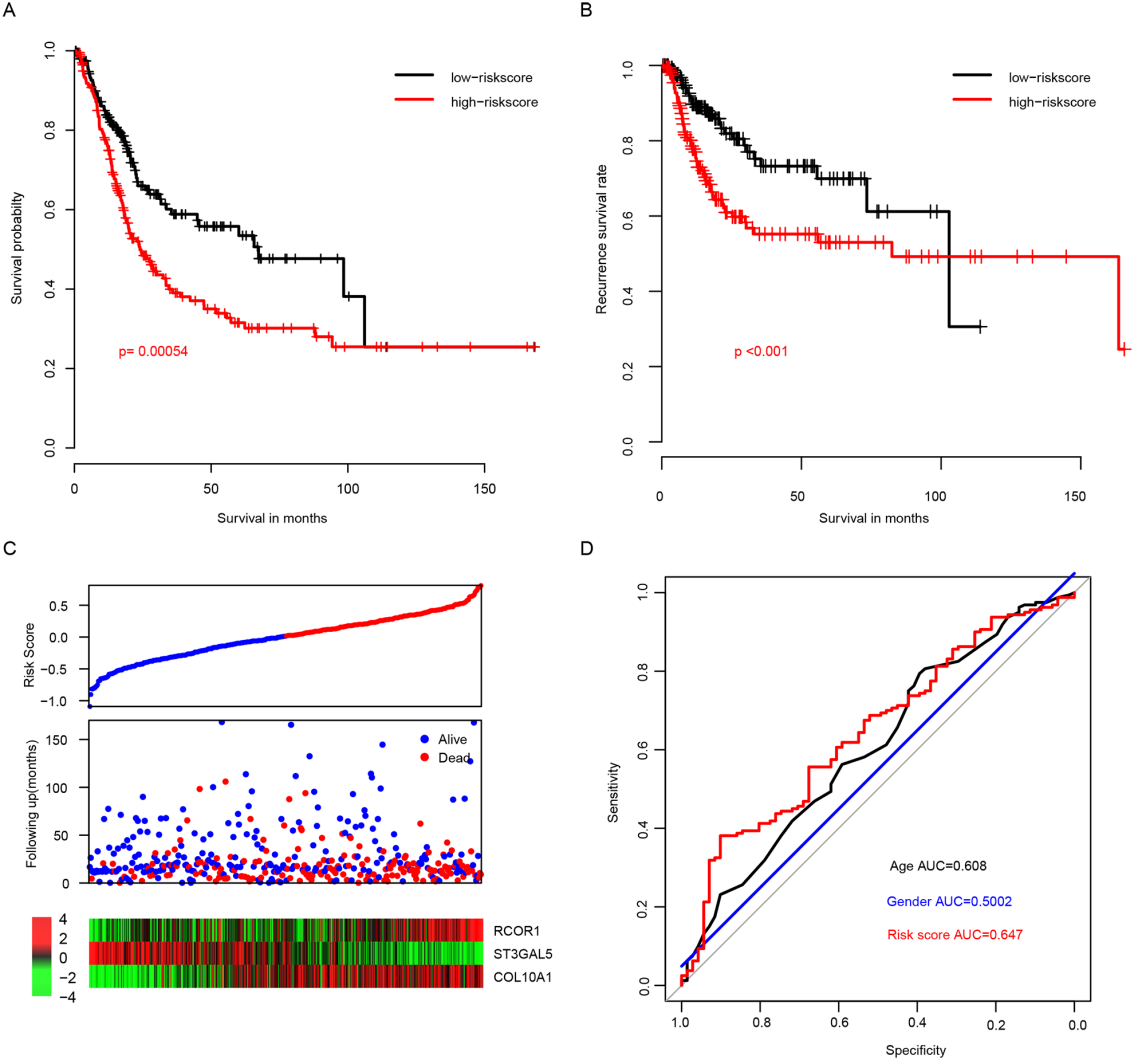
Performance of risk score in the training dataset (TCGA) The overall survival **(A)** and recurrence-free survival **(B)** rate in low-risk group is significantly higher than high-risk group, and the survival details were shown in **(C)** the three-year survival receiving operating characteristic curve (ROC) plotted and area under curves (AUC) were calculated **(D)**.

To measure the performance of risk score in predicting the outcome of BLCA patients, the survival of patients with high/low risk score were compared using the median risk score value as cutoff. The overall survival (OS) of patients with high risk score is significantly longer than those with low risk score (Figure 1A, p=0.00054). The median survival time of high risk group was 24 months and the median survival time of low risk group was 67.3 months. Re-sampling was also implemented by randomly retrieving 80% of all samples, and the possibility that risk score not significant associated with overall survival (p>0.05) was 0.0083 (Supplementary Figure 1). In addition, recurrence free survival (RFS) difference was also calculated between the high and low risk groups, and the result is consistent with the OS profile (Figure 1B, p<0.001). As shown in Figure 1C, patients in high risk score were characterized as early relapse, low expression of ST3GAL5, and high expression of RCOR1 and COL10A1. The Receiving operating characteristic curve (ROC) of three-year survival was also plotted according to age, gender, and risk score (Figure 1D), and the area under curve (AUC) was 0.608, 0.5002, and 0.647, respectively, indicating that the risk score performs better in predicting the survival of BLCA patients than other clinical information.

### Validation of performance of risk score in test datasets

**Figure 2:**
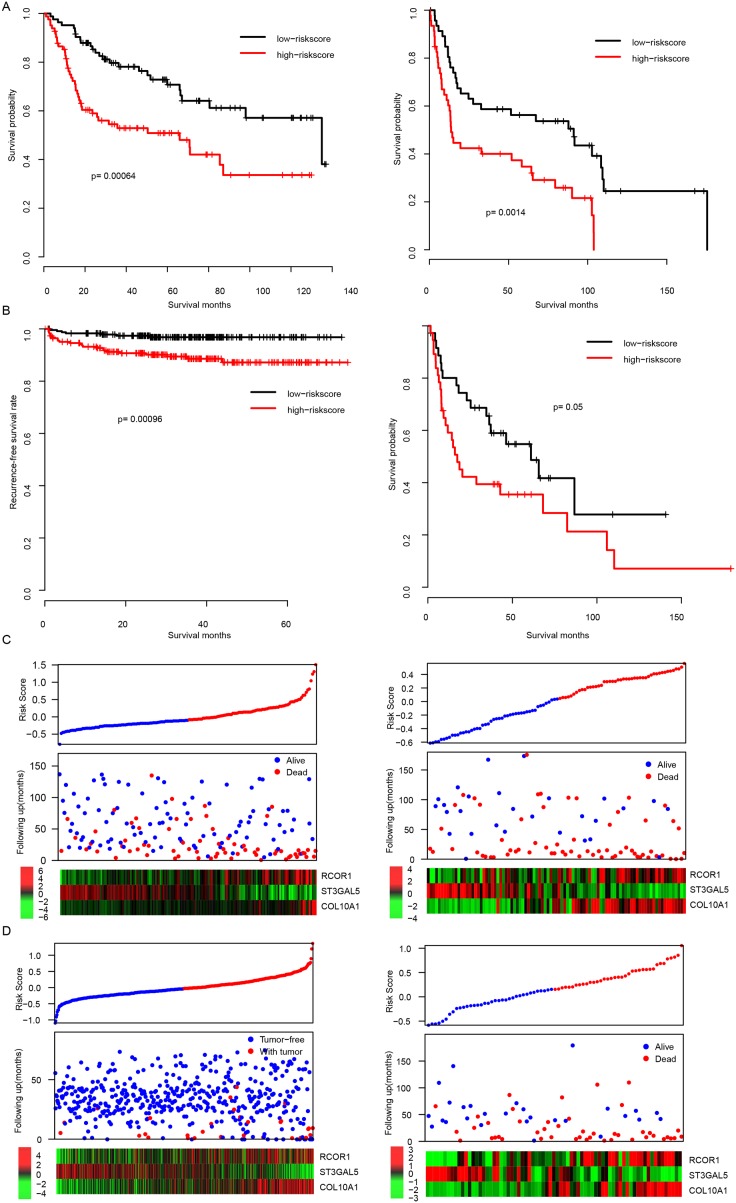
The performance of risk score in validation dataset The overall survival difference of high/low-risk group were shown in GSE13507 and GSE31684 datasets (**A**, left and right, respectively). Profiles of Recurrence-free survival and overall survival rate of another two totally independent datasets (E-TABM-4321 and GSE40875) were similar **(B)**. Detailed survival information was shown (**C** left for GSE13507, right for GSE31684, **D** left for E-TABM-4321 right for GSE40875).

To evaluate the robustness of our model, after locking the coefficients of each gene, the risk scores in another two independent datasets (GSE13507 and GSE31684) were evaluated. In consistent with the survival profile of the training dataset, the overall survival rate of high risk group is significantly lower than the low risk group in both datasets (p=0.00064 and 0.0014 for GSE13507 and GSE31684, respectively, Figure 2A). Since these two datasets were also used in candidate gene selection and over-fit may have brought, we used another two totally independent datasets (GSE48075 and E-TABM-4321) for further validation. Consistent with the observation in training datasets, early recurrence rate in E-TABM-4321 high risk samples was significantly higher than low risk samples. Similar trend was also observed in GSE48075 of overall survival rate (Figure 2B). In addition, the overall expression profiles of candidate genes used for risk score evaluation were also similar, compared to the training datasets (Figures 2C-2D). These results suggest that the risk score was robust in predicting the survival of BLCA patients.

### Relationship between risk score, other clinical information, and radiation

**Figure 3:**
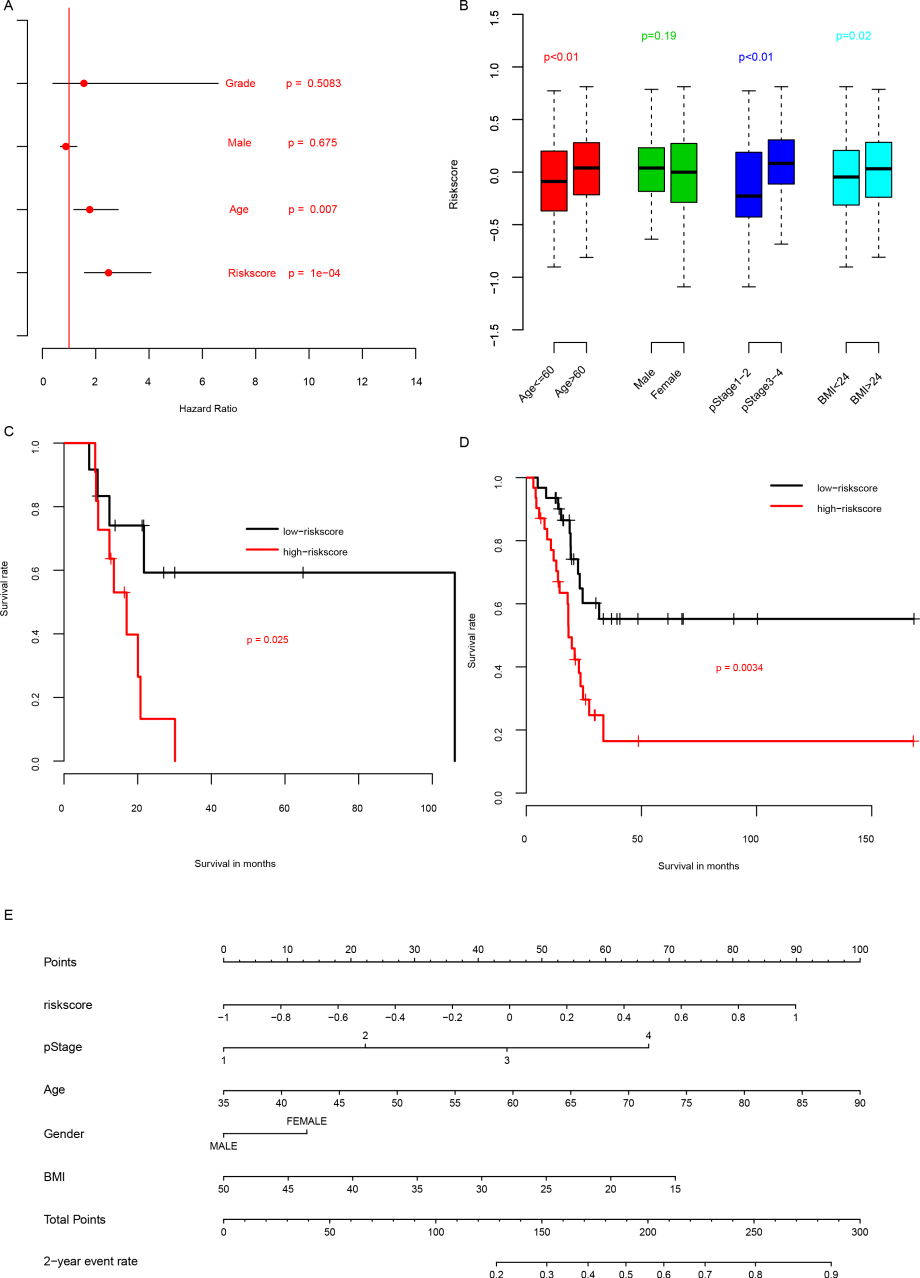
Clinical information and risk score The clinical significance of clinical information and risk score **(A)**, and association between them **(B)**. The performance of risk score on patients underwent radiation **(C)** and without radiation **(D)** was also plotted. A nomogram containing clinical information and risk score was plotted **(E)**.

In order to compare clinical significance of clinical observations and risk score, multivariate Cox hazard analysis was performed to evaluate the importance of these indicators. As shown in Figure 3A, the most important hazard factor for BLCA is risk score, while gender and histologic grade was not statistically significant. The correlation analyses between risk score and clinicopathological indicators showed that the risk score is significantly associated with age, primary tumor stage and BMI (body mass index), while independent from gender (Figure 3B).

Radiation is one of the most common therapy methods for BLCA. To evaluate whether risk score is also suitable for patients underwent radiation therapy, we artificially divided the patients underwent radiation therapy into high risk group and low risk group using median risk score as cutoff, as usual. The overall survival rate of patients underwent radiation therapy (Figure 3C) with high risk score had a significantly shorter survival rate than these with low risk score. And this trend was also repeated in the patients without radiation therapy (Figure 3D). To facilitate the utilization of risk score, a nomogram was plotted (Figure 3E). All these results above suggest that the prognostic performance of risk score is effective for both patients with and without radiation therapy.

### Altered pathways in the high risk score patients

**Figure 4:**
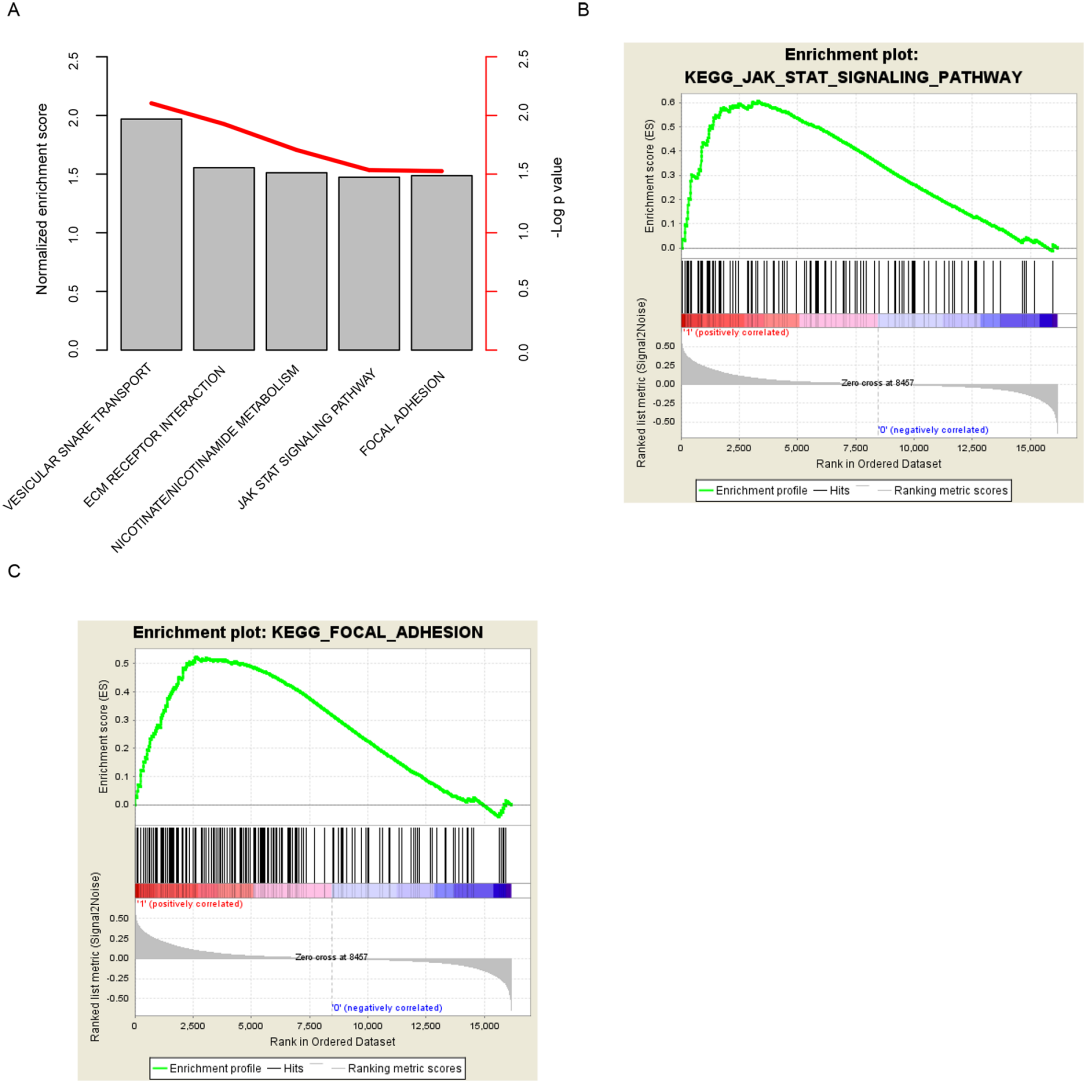
KEGG pathways associated with risk score The high-risk score associated pathways were calculated **(A)** with GSEA, and JAK-STAT signaling pathway **(B)** and focal adhesion **(C)** were noted.

The significantly altered signaling pathways between high risk group and the low risk group were assessed with Gene Set Enrichment Analysis (GSEA) to investigate why the risk score predicts the survival of BLCA patients. The significantly altered pathways in high risk group include “vascular SNARE transport”, “ECM receptor interaction”, “JAK-STAT signaling pathway”, and “focal adhesion” (Figure 4A, Supplementary Table 2). Among these KEGG pathways, “JAK-STAT signaling pathway” (Figure 4B) and “focal adhesion” (Figure 4C) associated genes were noted, suggesting that our risk score reflected the alteration of JAK-STAT signaling pathway and focal adhesion status, and thus predicting the prognosis of BLCA patients.

## DISCUSSION

The prognosis of BLCA patients is still difficult by clinical information, including TNM staging, age, etc. [5, 6] Thus, the molecular biomarker for prognosis is critically needed for treatment. In the past decades, although a lot of single molecular markers for prognosis have been reported [7–9], the clinical performance across datasets is not very satisfactory. On the other hand, multiple genes’ predicting effect has been highlighted [10–14]. In our current work, by using Cox multivariate regression on TCGA datasets, we report that the expression of three genes based risk score successfully predicted the survival of BLCA, and this finding is validated in four independent datasets. Totally, 1214 BLCA samples in 5 distinct datasets and two platforms involved in this study, and our model is effective in all of these datasets. Compared to other clinical information, the risk score contributed more, and performs better for survival predicting. In addition, the risk score predicting ability is robust for patients underwent radiation therapy or not.

During the last years, lots of single prognostic biomarkers for bladder cancer have been reported, including mRNAs, lncRNAs, and miRNAs, and clinical significantly associated genes were identified. For example, HMGA2 was found to be associated with epithelial-to-mesenchymal transition in bladder cancer [15], and SKIP was reported to be associated with histological grades and poor prognosis [16]. Up-regulation of miRNAs including miR-141 [17] and miR-34a [18] often indicates a favorable survival. LncRNAs were also reported to predict survival of bladder cancer [19, 20]. However, the clinical utilization of these single biomarkers still need more investigation. One of the evidences is that, none the aforementioned mRNAs are not significantly associated with survival in our datasets. For example, HMGA2 was statistically significantly associated with overall survival only in TCGA dataset (p=0.022), but not GSE13507, GSE31684, GSE48075 and E-TABM-4321 (p=0.0898, 0.476, 0.0553 and 0.183 respectively, data not shown). On the other hand, multiple gene expression considered more information and thus more robust in prognosis, as previous reports [21–26]. Our results showed that the risks score performs better in 1214 samples consist of five independent datasets, consistent with these reports.

Of these three genes, RCOR1 interacts REST (RE1-silencing transcription factor) and modulate chromatin structure together with REST [27]. The prognostic effect of RCOR1 has been elucidated across cancer types, including glioma [28], and diffuse large B-cell lymphoma [29], although the prognostic effect of RCOR1 is still vague in bladder cancer. Another gene, ST3GAL5, has been reported to be positively correlates with the high risk of pediatric acute leukemia [30, 31] and associated with multidrug resistance in human acute myeloid leukemia, indicating the function of ST3GAL5 in carcinogenesis and development. The third gene, COL10A1, has been widely reported to be a oncogene in multiple cancer types [32], contribute to vasculature, and also used as biomarker for neo-adjuvant therapy effect prediction indicator in ER+/HER2+ breast cancer [33]. All the genes except for ST3GAL5 used were oncogenes according to the risk score formula. The positive coefficients of these genes in the risk score formula suggest that these genes contribute to the risk score and thus predict poor survival of bladder cancer, which is consistent with the aforementioned reports, except for ST3GAL5. It is considered that this may result from the heterogeneity among cancers. We also noticed that the functions of genes involved in this study were different, which may explain why the robustness of risk score is better than single gene biomarkers.

The significantly altered pathways include focal adhesion, and other related KEGG pathways, suggesting that the risk score reflected the cell-cell interaction status of BLCAs.

## MATERIALS AND METHODS

### Data pre-processing

The TCGA dataset were downloaded from UCSC Xena website (http://xena.ucsc.edu/), the expression value were converted to normalized RSEM values, the detailed pre-processing steps, including mapping and normalization, were described on UCSC Xena website. Genes expressed in less than 80% samples were discarded, and for 0 values were replaced with 1/2 of the minimum RSEM value except for 0 values of the corresponding gene. The expression matrix was then transformed with log 2.

Raw data of GSE13507, GSE31684, GSE48075, and E-TABM-4321 was downloaded in. CEL format from GEO (https://www.ncbi.nlm.nih.gov/geo) and array expression (www.ebi.ac.uk/arrayexpress/). After background correction, and normalization with Robust Multiarray Averaging (RMA) using R package “affy” function rma(), probes was mapped to gene name based on the manufacture provided annotation file. Genes matching more than one probe were merged and average values were calculated as their expression values.

### Prediction gene selection and cox multivariate regression model

Cox univariate regression were performed in TCGA, GSE13507, and GSE31684 datasets to select the survival-related genes. Gene significantly associated with overall survival (p<0.05) in all of these datasets were retained for further analyses, and three genes were selected. Multivariate Cox Regression was performed to develop the risk score staging model with the candidate genes using R package “survival” function coxph(), and coefficients were locked for other datasets. The formula of risk score is described as the following,

$$\mathrm{Risk score}=\sum_{i}^{n}\beta_{i}*x_{i}$$

Where β_i_ indicates the coefficients of genes and x_i_ refers to the relative expression of corresponding gene. The coefficients of β_i_ was calculated in the TCGA datasets and locked for assessing the risk score of samples in the other four independent datasets. Median risk score was used as cutoff values in discriminating the high and low risk group, and the survival difference was compared with Kaplan survival plot.

### Statistical analysis

All statistical analysis was carried out with R (https://www.r-project.org/, v3.0.1) and R packages. Normalization of affymetrix raw data was performed with R package “affy”. The survival analysis and cox probability hazard model development was implemented with R package “survival”. The ROC curves were plotted with R package “pROC” [13], and nomogram was drawn with R package “rms”. The Gene Set Enrichment Analysis was performed with java software GSEA (http://software.broadinstitute.org/gsea/index.jsp) [34].

## SUPPLEMENTARY MATERIALS FIGURE AND TABLE


