# Supplementary material for: Risk score based on three mRNA expression predicts the survival of bladder cancer
Source: Oncotarget. 2017 Jun 27;8(37):61583–91. doi: 10.18632/oncotarget.18642 (PMC5617447; doi:10.18632/oncotarget.18642)
Supplement: Supplementary file 1 [file oncotarget-08-61583-s001.pdf]

Risk score based on three mRNA expression predicts the survival of bladder cancer

SUPPLEMENTARY FIGURE AND TABLES

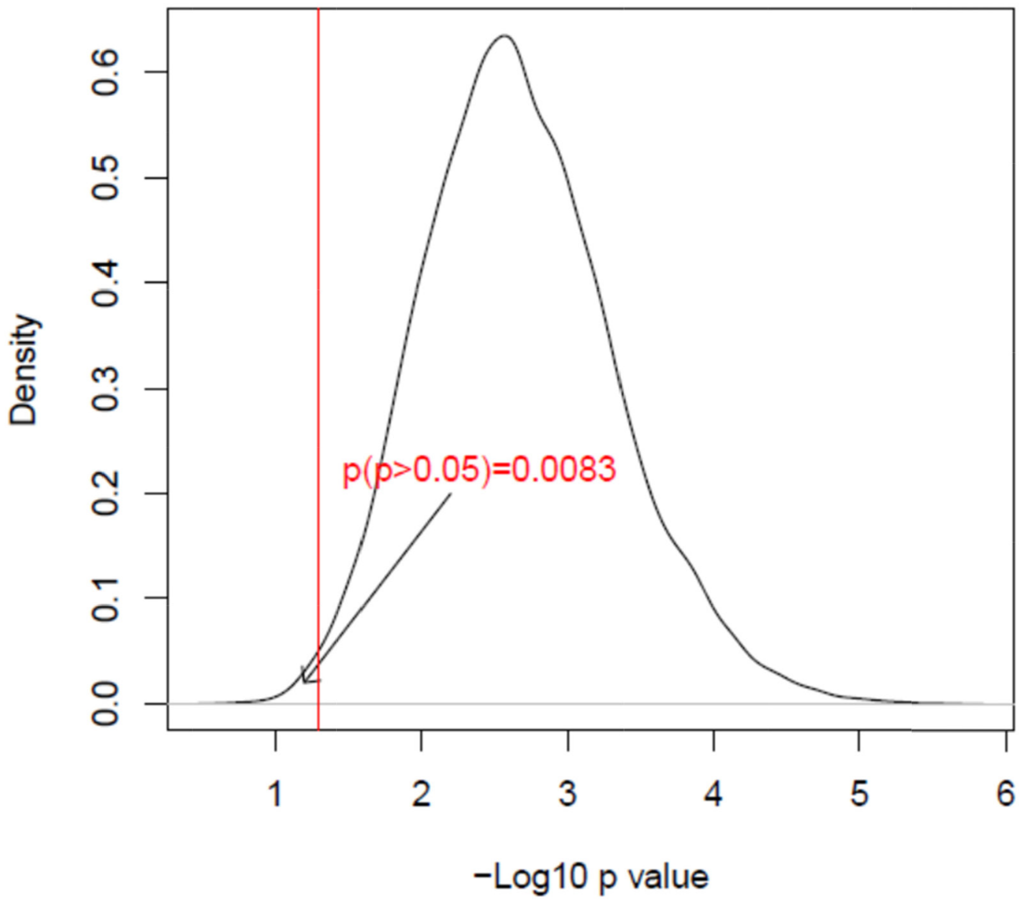

Supplementary Figure 1: Distribution of P values generated from comparing survival of high/low risk group of 80% Resampling samples for 10000 times. The possibility of not significant (p>0.05) was 0.83%.

Supplementary Table 1: The parameters of the three selected genes in univariate regression and multivariate regression

Gene	Univariate regression			Multivariate regression		
	HR	95% CI	p Value	HR	95% CI	p Value
RCOR1	1.259	1.085-1.461	0.0024	1.2	1.031-1.396	0.018634
ST3GAL5	0.772	0.666-0.895	0.0006	0.841	0.715-0.989	0.03686
COL10A1	1.199	1.024-1.403	0.0243	1.159	0.978-1.373	0.088168

HR refers to hazard ratio; 95% CI is 95% confidence interval.

Supplementary Table 2: The details of GSEA analysis

Name	SIZE	NES	p-Value
SNARE_INTERACTIONS_IN_VESICULAR_TRANSPORT	38	1.969208	0.0079
ENDOMETRIAL_CANCER	50	1.608377	0.0079
CHRONIC_MYELOID_LEUKEMIA	73	1.629621	0.0095
GLYCOSAMINOGLYCAN_DEGRADATION	19	1.657602	0.0101
ECM_RECEPTOR_INTERACTION	79	1.554296	0.0118
NICOTINATE_AND_NICOTINAMIDE_METABOLISM	22	1.511451	0.0197
GLIOMA	64	1.546252	0.0213
JAK_STAT_SIGNALING_PATHWAY	113	1.4731	0.0292
AMINO_SUGAR/NUCLEOTIDE_SUGAR_METABOLISM	43	1.62112	0.0296
FOCAL_ADHESION	189	1.486739	0.0298
PANCREATIC_CANCER	70	1.494939	0.0369
CYTOKINE_CYTOKINE_RECEPTOR_INTERACTION	204	1.403009	0.0398
PYRIMIDINE_METABOLISM	95	1.516201	0.0451
NOD_LIKE_RECEPTOR_SIGNALING_PATHWAY	58	1.508246	0.0453
SYSTEMIC_LUPUS_ERYTHEMATOSUS	99	1.436914	0.0457

The Name is KEGG pathways, NES indicates the normalized enrichment score.
